# Free to Circulate: An Update on the Epidemiological Dynamics of Porcine Circovirus 2 (PCV-2) in Italy Reveals the Role of Local Spreading, Wild Populations, and Foreign Countries

**DOI:** 10.3390/pathogens9030221

**Published:** 2020-03-17

**Authors:** Giovanni Franzo, Susanna Tinello, Laura Grassi, Claudia Maria Tucciarone, Matteo Legnardi, Mattia Cecchinato, Giorgia Dotto, Alessandra Mondin, Marco Martini, Daniela Pasotto, Maria Luisa Menandro, Michele Drigo

**Affiliations:** Department of Animal Medicine, Production and Health (MAPS), University of Padua, 35020 Legnaro, PD, Italy

**Keywords:** PCV-2, Italy, genotyping, domestic pig, wild boars, molecular epidemiology

## Abstract

Porcine circovirus 2 (PCV-2) is one of the most impactful and widespread pathogens of the modern swine industry. Unlike other DNA viruses, PCV-2 is featured by a remarkable genetic variability, which has led to the emergence and recognition of different genotypes, some of which (PCV-2a, 2b, and 2d) have alternated over time. Currently, PCV-2d is considered the most prevalent genotype, and some evidence of differential virulence and vaccine efficacy have been reported. Despite the potential practical relevance, the data on PCV-2 epidemiology in Italy are quite outdated and do not quantify the actual circulation of this genotype in Italy. In the present study, 82 complete ORF2 sequences were obtained from domestic pigs and wild boars sampled in Northern Italy in the period 2013–2018 and merged with those previously obtained from Italy and other countries. A combination of phylogenetic, haplotype network, and phylodynamic analyses were used to genotype the collected strains and evaluate the temporal trend and the spatial and host spread dynamics. A rising number of PCV-2d detections was observed in domestic pigs, particularly since 2013, reaching a detection frequency comparable to PCV-2b. A similar picture was observed in wild boars, although a lower sequence number was available. Overall, the present study demonstrates the extreme complexity of PCV-2 molecular epidemiology in Italy, the significant spread across different regions, the recurrent introduction from foreign countries, and the frequent occurrence of recombination events. Although a higher viral flux occurred from domestic to wild populations than vice versa, wild boars seem to maintain PCV-2 infection and spread it over relatively long distances.

## 1. Introduction

*Porcine circovirus 2 (PCV-2)*, a member of the genus *Circovirus* and family *Circoviridae*, is one of the most relevant pathogens for the swine industry [1]. Initially isolated in the middle of the 1990s from pigs affected by post-weaning multisystemic wasting syndrome (PMWS) [2,3], it was then rapidly reported all around the world [4,5] and associated with several syndromes (currently called Porcine circovirus diseases; PCVD) including reproductive disorders, the so-called porcine respiratory disease complex (PRDC), enteritis, porcine dermatitis and nephropathy syndrome (PDNS), and proliferative and necrotizing pneumonia (PNP) [6]. However, PCV-2 is frequently detected in healthy animals even though productive losses have been demonstrated in subclinically infected animals [7].

Interestingly, the first retrospective evidence of PCV-2 infection was found in German archive samples collected in 1962 [8], and the origin of this virus was further backdated by phylodynamic analysis to the beginning of the past century [9]. Its recent emergence as a major swine pathogen suggests the remarkable role of intensive swine farming in affecting PCV-2 epidemiology [9]. While swine is the primary host for PCV-2, other species have been reported as susceptible [10]. Nevertheless, wild boar seems to be the only other host in which PCV-2 is constantly detected and which could play a relevant role in PCV-2 epidemiology [11,12,13,14].

The viral genome consists of a single circular strand of DNA of approximatively 1.7 kb containing two main Open Reading Frames (ORFs): ORF1, coding for Rep and Rep’ proteins involved in viral replication; and ORF2, encoding Cap, the sole component of the viral capsid [15]. Additionally, other ORFs and related proteins (ORF3-6) have been characterized and proven to be involved mainly in apoptosis regulation and interaction with the host immune system [16,17,18,19].

As other ssDNA viruses, PCV-2 features a remarkable evolutionary rate, due to both high mutation and recombination rates, which has led to the development of significant genetic heterogeneity [5,20]. Currently, eight genotypes have been formally defined based on ORF2 phylogenetic analysis [5]. However, only three of them (PCV-2a, -2b, and -2d) are widespread and have alternated over time. PCV-2a was the most common one until approximatively 2003, when it was replaced by PCV-2b [21,22,23]. Since approximatively 2010, a new shift seems in place with the rise of PCV-2d [9,20,24].

Despite the relevance of this pathogen, available information on the Italian epidemiological scenario is scarce and outdated (i.e., until 2014) [25]. At that time, PCV-2b was by far the most prevalent genotype, and no clear evidence of the shift to PCV-2d was present in this country. A recent study evaluating PCV-2 presence in Sardinian domestic pigs and wild boars revealed an increase in PCV-2d frequency [26]. It should be noted that Sardinia is extremely peculiar given the type of farming (mainly free-ranging and producing piglets for immediate human consumption) and the trade restrictions with the rest of the world due to the presence of African Swine Fever (ASF) [27].

For these reasons, Sardinia cannot be considered representative of the overall Italian scenario. Therefore, an update of PCV-2 strains circulating in Italy, including information on the highly dense populated area of Northern Italy, is mandatory. Additionally, comparison of PCV-2 genetic data in domestic pigs and wild boars located in these two areas could help in elucidating the impact of such different production systems on PCV-2 epidemiology.

## 2. Results

Sixty-nine complete ORF2 sequences were obtained from Italian domestic pigs: one was classified as PCV-2a, 39 as 2b, and 25 as 2d. Additionally, four recombinant strains were identified, likely originating from three different recombinant events. Recombinant strains are listed as follows:

- strain 9607 originated from a recombination event between PCV-2d (region 0–350) and PCV-2a (region 351–699);
- strain 16860 between PCV-2b (region 0–105 and 270–699) and PCV-2d (region 106–269);
- strains 9473 and 13719 between PCV-2d (region 0–312) and PCV-2b (region 313–699).

Sixty-seven out of 122 samples (54.9%) from wild boars tested positive for PCV-2. However, most of them displayed low to very low viremia (median = 25.33 copies/mL), and only 13 complete ORF2 sequences could be obtained. Of those, one strain was classified as PCV-2a, nine as PCV-2b, and three as PCV-2d. No recombinant sequences were identified in wild animals. All sequences have been submitted to GenBank (Acc. Number MT068214–MT068295).

**Figure 1:**
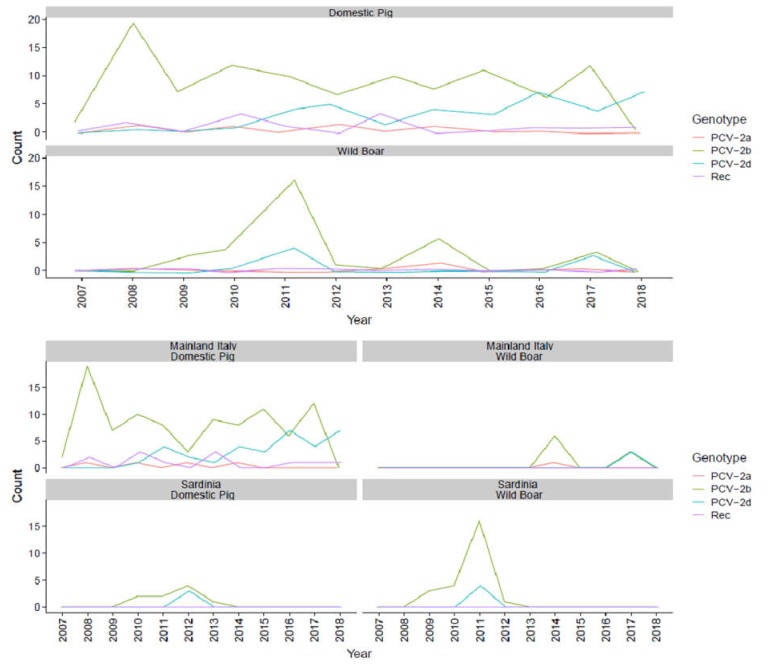
Temporal trends reporting the count of each genotype detection. Recombinant count has also been included. In the lower figures, counts are further classified based on their collection from Sardinia (island) or mainland Italy.

The genotype trend over time was evaluated after merging the sequences obtained in the present study with those reported in Franzo et al. (2015) [25] and Dei Giudici et al. (2017) [26]. Strain classification was re-evaluated according to the updated classification criteria, and recombination analysis was performed on the new dataset. The obtained results were consistent with the previous studies. In Figure 1, the counts of PCV-2a, 2b, and 2d and the recombinant strains are reported over time.

In domestic pigs, PCV-2b remained the most frequently detected strain during the whole study period and showed an essentially stable frequency. PCV-2a detections were rare and sporadic until 2015 when this genotype became undetectable. On the contrary, PCV-2d occurrence increased from 2010, reaching a frequency comparable to PCV-2b in 2016. The only significant exception was 2018, when only PCV-2d was reported. Recombinant strains were detected at low frequencies throughout the study (Figure 1). A comparable scenario was observed in wild boars: PCV-2b was the most frequently reported genotype over the whole study duration, while PCV-2a was detected only once in 2014, and PCV-2d was reported in 2011 (n = 4 detections) and 2017 (n = 3 detections).

**Figure 2:**
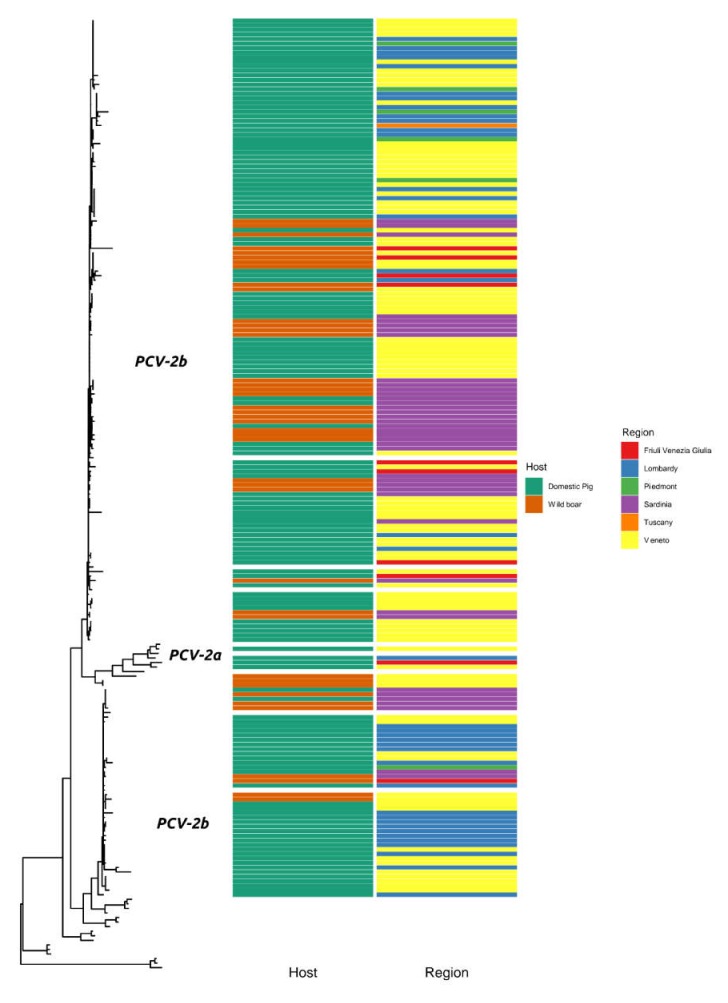
Maximum likelihood phylogenetic tree based on the ORF2 of Italian strains collected from wild boars and domestic pigs. The host and region of origin are color coded in the rectangles nearby the corresponding tip.

Even if small sequence clusters tended to include strains collected in the same host or region (Figure 2), the overall tree evaluation did not highlight any clear geographic or host clustering among Italian strains. In fact, groups of sequences collected from wild boars were well distributed in the phylogenetic tree. Similarly, strains collected in different regions were widely interspersed in the phylogenetic tree.

**Figure 3:**
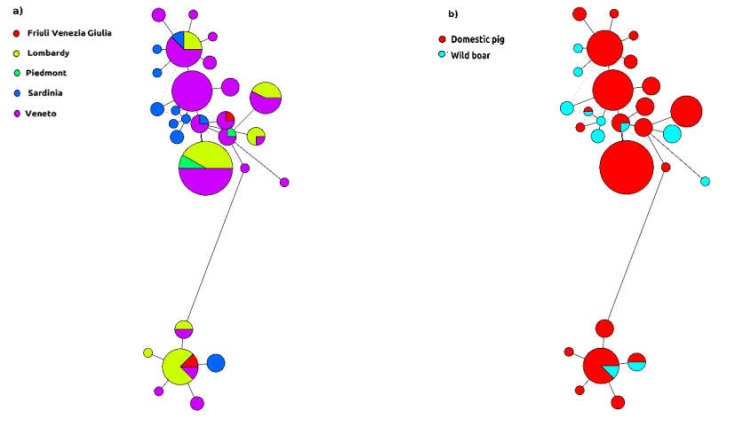
Haplotype network of Italian Porcine circovirus 2 (PCV-2). The circumference is proportional to the sequence number, while geographical distribution (**a**) and collection host (**b**) are represented with different colors. For graphical reasons, only haplotypes including more than 2 strains have been included.

This pattern was confirmed by haplotype network analysis (Figure 3). Strains collected in different regions were frequently classified within the same haplotype (or in closely related ones), although all haplotypes are featured by a “dominant” region. Accordingly, the same region harbored different haplotypes (Figure 3a). A similar picture can be drawn for the host, since several haplotypes included both wild and domestic animals. Even though some haplotypes including only wild boars were also demonstrated, they were genetically highly related to others comprising domestic pig sequences (Figure 3b). The estimation of strain exchange among hosts revealed an asymmetric directionality of the viral flux, with a migration rate approximatively three times higher (i.e., 1.41 vs. 0.53) from the domestic to wild population than from the wild to domestic one.

**Figure 4:**
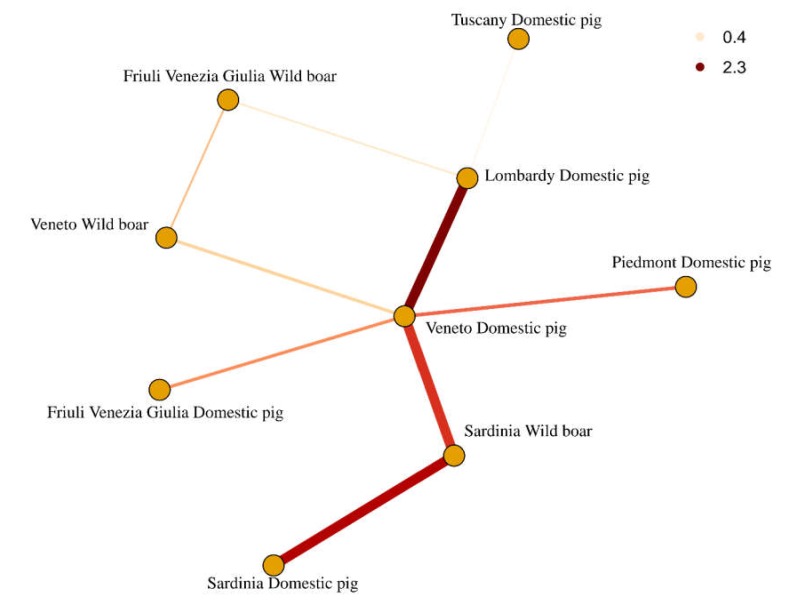
Network reporting the well-supported transmission path (Bayesian factor (BF) > 3) among Italian region–host pairs estimated using the discrete trait reconstruction approach implemented in BEAST 1.8.4. The line size is proportional to the BF value, and the transmission rate is color coded.

The reconstruction of viral migration among Italian host–region pairs demonstrated a dense network of contacts. While intense and statistically significant migration rates were identified mainly between domestic pig populations of different regions, even wild ones were significantly involved. Contacts between wild populations (e.g., wild boars of Friuli Venezia Giulia and Veneto) and also between wild and domestic populations (e.g., Veneto wild boars and domestic populations, Friuli Venezia Giulia wild boars and Lombardy domestic pigs, as well as Sardinia wild boars and Sardinia and Veneto domestic pigs) were also inferred (Figure 4).

**Figure 5:**
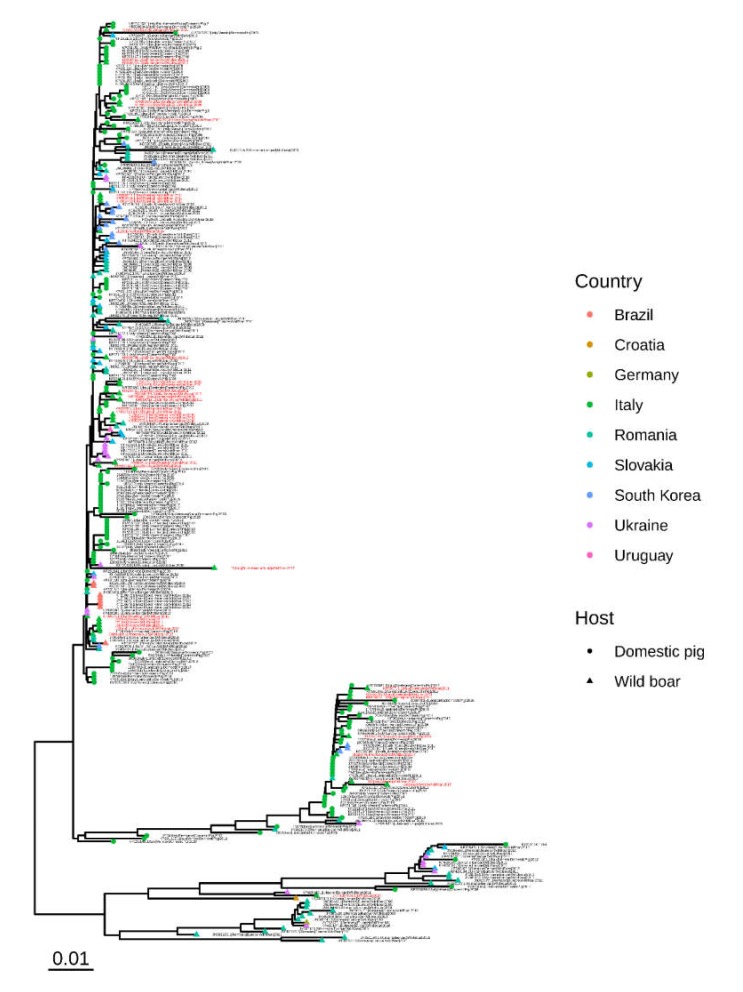
Maximum likelihood phylogenetic tree based on the ORF2 of all Italian strains plus those collected from wild boars all around the world. The host and collection country are represented with different shapes and colors, respectively. Italian wild boar sequences are highlighted in red.

Comparison with worldwide collected sequences revealed Italian ones were distributed in the overall tree and mixed with strains collected all over the world, although some monophyletic Italian clusters were identified (Supplementary Figure S1). Interestingly, Italian wild boar strains were more closely related to other Italian domestic/wild boar sequences than to others obtained from worldwide sampled wild boars (Figure 5).

## 3. Discussion

Even though swine production plays a major role in Italian farming, the epidemiological knowledge of such an impacting pathogen is patchy and largely outdated. In previous studies, PCV-2b was the most frequently detected genotype in major Italian swine production regions. The rise of PCV-2d, although firstly identified in 2010 and confirmed to circulate in Sardinia domestic and wild boars, was not observed in the intensive farming sector [25,26].

The present study confirms the presence of only three major genotypes—PCV-2a, 2b, and 2d—and demonstrates that an increase in PCV-2d also occurred in Italy after 2013.

Differently from what was reported on a worldwide scale [9,20,28], a proper genotype replacement (genotype shift) cannot be stated. In fact, both PCV-2b and 2d seem to circulate in the Italian population at comparable frequencies. The causes behind the increase in PCV-2d frequency are still unknown. Experimental reports about a higher PCV-2d virulence are contradictory [29,30,31], likely because of the multifactorial nature of PCVD. More consistent evidence is present regarding differential cross-protection among strains belonging to different genotypes due to the variability in epitopic domains [9,32,33,34]. While the efficacy of currently available PCV-2a-based commercial vaccines to protect against clinical sign onset was demonstrated in several experimental trials [30,35,36], differences have been highlighted in terms of immune response and viremia. In fact, homologous vaccines can guarantee a better (or complete) viremia control and higher neutralizing antibodies [31,37,38] compared to heterologous ones. PCV-2 vaccines have sometimes been considered a “leaky vaccine” [36], meaning they tend to decrease transmission and infection rates in controlled situations such as in a vaccine trial or in the presence of good farm conditions, but they may not confer full protection under conditions of repeated exposure and in the presence of other cofactors [39]. It is, therefore, possible to speculate that the observed PCV-2d rise could be at least partially due to suboptimal cross-protection, which could be magnified in field conditions where errors in vaccine management and timing, immunosuppression, and progressive waning of immunity are likely to occur.

Why the second genotype shift remained partial in Italy is similarly obscure. Italian swine farming has some peculiarities compared to other countries, since pigs are raised until about 160 kg for the production of cured ham. The slower population turnover, the vanishing of vaccine immunity in aging animals, and the unavoidable management complications (e.g., difficulties in all-in all-out, flow management, etc.) could affect PCV-2 epidemiology, enhancing the persistence of some strains, as previously suggested for Porcine reproductive and respiratory syndrome virus (PRRSV) [40]. Actually, a significant proportion of PCV-2d success in Italy could be due to the import of foreign strains. In fact, PCV-2d presence in Italy is the result of multiple introduction events, as supported by multiple clades closely related to strains collected in other regions (Supplementary Figure S1). Thus, the observed rise in PCV-2d frequency reflects a worldwide increase of this genotype, coupled with the limited efficacy of biosecurity measures preventing new strain introduction. A relatively similar topology also affects PCV-2b, although bigger Italian-only clades can be observed, suggestive of a prolonged circulation in the field as previously reported [25].

The absence of any effective compartmentalization among regions was confirmed, evidencing a likely weak spot of Italian swine faming (and of other sectors as well [41]) that has not improved over time. In addition to the intrinsic challenges in the diagnosis of an often subclinical infection, poorly effective biosecurity measures and the vertical integration of swine production, featured by a limited number of sow farms providing piglets to multiple growers and finishers located in multiple regions, likely play roles in the observed scenario. Recombination events were constantly detected during the whole considered period. Besides confirming PCV-2 propensity to recombine [42], these findings demonstrate that co-infections with multiple genotypes are not rare and likely are due to management practices and frequent animal mixing [43]. All detected events were single identifications or small clusters very limited in time and space, suggesting the low fitness of these recombinant variants. Nevertheless, the emergence of high-fitness recombinant strains has also been demonstrated [9]; therefore, more effort should be aimed at limiting the risk of co-infection and recombination occurrence.

Interestingly, even Sardinia, an island featured by monodirectional swine trade and with a marginal involvement in the framework of intensive swine farming, shares haplotypes with Northern Italy, suggesting that the limited animal importation from the Italian mainland is effectively contributing to PCV-2 epidemiology.

The presence of PCV-2 in wild boars was also investigated. A high animal percentage (more than 50%) tested positive for PCV-2, confirming an actual role of wild boars as reservoirs of PCV-2 in nature [44].

The significant circulation of PCV-2 in wild animals was already proven, even though with a typically lower prevalence. Part of the samples tested in the present study were from the Colli Euganei Regional Park, a relatively small area with high animal densities, which could justify the high viral prevalence observed in these samples as suggested by Vicente et al. (2004) [44]. However, a similar PCV-2 frequency was observed in samples originating from Friuli Venezia Giulia, where the animal density was lower by several orders of magnitude. These results are comparable with those obtained for PCV-3 in the same areas [45,46], suggesting the capability of PCVs to spread and persist in wild boars regardless of the animal density. Interestingly, just 1 out of 12 samples originating from the Belluno Dolomites, an environment with features comparable to Friuli Venezia Giulia, tested positive. Therefore, other unconsidered factors are likely involved in PCV-2 epidemiology in wild animals and deserve further investigation. Nevertheless, the typically low viral titers lessen its clinical relevance in wild animals.

The absence of a clear host-clustering testifies frequent strain exchange between wild and domestic animals, as previously reported [46]. This pattern is confirmed by wild boar strains being often part of clusters including domestic pig sequences collected in the same region. While this scenario could be expected in Sardinia, where many small family-operated farms are present, it is much harder to explain in Northern Italy, where biosecurity measures of intensive farming should prevent contact between wild and domestic animals. The relationship between wild and domestic animals has been long discussed for circoviruses. The performed discrete trait (i.e., host) analysis highlighted the asymmetric directionality of the viral flux, with a migration rate approximately three times higher from domestic pigs to wild boars than vice versa. Therefore, based on the obtained results, the “who threatens who” dilemma can be solved in favor of the wild population, which appears to be the more endangered population, rather than a danger for the swine industry. Surely, more extensive epidemiological studies are required to confirm these results, detect the transmission route and evaluate the real impact on wild animal health and ecology.

A potential bias in our estimations can be due to the presence of other countries (not considered in the present study because of the limited sequence availability and increasing computational complexity) that could be involved in the viral exchange between the Italian wild and domestic swine populations. Although our assumption can be strong, it appears reasonable and supported by the data. In fact, all Italian wild boar sequences were genetically part of (or closely related to) haplotypes including other Italian sequences. Thus, a parsimony criterion suggesting an essentially within-country spreading can be advocated. The lower number of sequences obtained from wild boar compared to domestic pigs could potentially affect estimations of the viral flux directionality. However, it must be stressed that the ratio between hosts (i.e., approximately 1:3) is not disproportionate, and the migration rates herein reported are not simply the result of a host transmission event count. On the contrary, the estimations are based on a more complex probabilistic method accounting for viral genealogy and ancestral relationships among strains, making this approach more robust to a certain imbalance in the features of sampled strains. Nevertheless, the acquisition of a higher number of representative PCV-2 sequences from wild boars could be of benefit in strengthening the present conclusions and evaluate their validity in other countries.

The presence of sequence clusters collected from wild boars at different time points evidences the ability of PCV-2 to persist in these populations independently of new introductions from domestic pigs. Additionally, detection of identical (or almost identical) wild boar strains in different Italian regions can be considered suggestive of the role of wild hosts in mediating viral spread over relatively long distances. These results were also confirmed by the inferred migration rates between wild and domestic populations located in different regions of Northern Italy (Figure 4). More surprisingly, the contact between domestic pigs in the mainland and Sardinia seems mediated by the Sardinia wild boar population. While direct contact between the Veneto domestic pigs and Sardinia wild boars can be excluded, wild boars could have acquired the infection from unsampled, rurally reared animals, potentially acquired from the mainland, and thereafter acted as a viral shedding factor among other rural farms located on the island. Interestingly, the importation of runts from intensive farms in Northern Italy to Sardinia rural ones, aimed at the production of traditional dishes, is a relatively common practice. The combination of lower health conditions of transported animals (potentially due to chronic infectious diseases like PCV-2), limited biosecurity measures implemented in family-operated farms and relative ease of wild–domestic animal contacts in these settings could have favored the introduction of new viral strains.

However, the limited number of available sequences also prevents definitive conclusions in this case.

The present study demonstrates a progressive rise of PCV-2d frequency in Italy over time. The epidemiological scenario was confirmed to be extremely complex in this country and was featured by the presence of all major genotypes, frequent recombination events, and an essentially unconstrained viral migration both from foreign countries and among Italian regions. Additionally, a high infection prevalence was observed in the wild boar population, which was significantly connected to the domestic pig one, apparently acting more as a recipient than as a source of strain introductions. Nevertheless, a non-negligible flux in the opposite direction was also estimated. Overall, these results highlight the limited efficacy of biosecurity-based control measures. While vaccines can be effective in controlling clinical signs, the vaccination-related direct and indirect costs, the emergence of new PCV-2 strains and genotypes and the likely role of vaccine immunity in driving viral evolution evidence that significant benefits could derive from implementing more effective measures to prevent, or at least limit, PCV-2 infection and spread.

## 4. Material and Methods

### 4.1. Italian Samples

The one hundred and twenty-two PCV-2-positive samples included in the present study originated from routine diagnostic activities of private laboratories who received samples from swine farms mainly located in the highly dense populated area of Northern Italy. All samples, including sera, lymph nodes, and lungs, were screened for PCV-2 by commercial real-time PCR assays, and the positives were delivered to the infectious disease laboratory of the Department of Animal Medicine, Production and Health (MAPS), University of Padua, for further molecular characterization. DNA was extracted from 200 µL of tissue homogenate or 200 µL of serum using the DNeasy Blood and Tissue kit (Qiagen) according to the manufacturer’s instructions. Samples and extracted DNA were stored at −20 °C until processing.

Additionally, 122 wild boar sera were included in the study. Some were archived samples originating from Colli Euganei Regional Park [45] and Friuli Venezia Giulia region [46], while additional ones were collected from Veneto (Belluno Dolomites) in 2017–2018 using the sampling approach described in Franzo et al. (2019) [46]. All considered swine samples were collected in the context of routine diagnostic activities, and no experimental treatments or additional assays were implemented during the study; likewise, game animals were hunted during the licensed hunting season and not specifically for this study. Therefore, in both cases, approval of the ethics committee was not required. DNA was extracted as previously described and PCV-2 presence investigated using an in-house designed real-time PCR. Briefly, the DyNAmo Flash Probe qPCR Kit (Thermo Fisher Scientific, Waltham, MA, USA) was used according to the following protocol: 2 μL of extracted DNA was added to a standard mix composed of 1 × DyNAmo Flash Probe qPCR master mix, 0.6 μM, and 0.3 μM of PCV-3–specific primers (P1570F 5’-TGGCCCGCAGTATTCTGATT-3’ and P1642R 5’-CAGCTGGGACAGCAGTTGAG-3’) and probe (P1591 5’-6FAM-CCAGCAATCAGACCCCGTTGGAATG-IBFQ-3’) [47], respectively. Sterile NANO-pure water was added to bring the final volume up to 10 μL. The cycling conditions were 95 °C for 7 min, followed by 45 cycles of 95 °C for 10 s, and 60 °C for 30 s. The fluorescence signal was acquired at the end of each cycle extension phase.

Amplification of ORF2 of all positive samples was attempted using two overlapping PCRs (PCR-C and -D) following the protocol described by Franzo et al. (2015) [25]. Positivity and specificity of the bands were verified by SYBRsafe stained agarose gel electrophoresis, and amplicons were Sanger-sequenced in both directions using the same primers as for PCR at Macrogen Europe (Amsterdam, The Netherlands). Chromatogram quality was evaluated using FinchTV (http://www.geospiza.com), and consensus sequences were assembled with ChromasPro (ChromasPro Version 2.0.0, Technelysium Pty Ltd, South Brisbane, Australia).

### 4.2. Sequence Analysis

Obtained sequences were trimmed to include ORF2 only and aligned to the reference dataset suggested by Franzo and Segalés (2018) [5] for genotyping. To account for the coding nature of ORF2, alignment was performed at the amino acid level using the MAFFT [48] method, and nucleotide sequences were then superimposed using TranslatorX [49].

Recombination analysis was performed using RDP4 [50] selecting RDP, GENECONV, MaxChi, and 3Seq methods for a primary scan, while all implemented methods were used to refine recombination detection. Settings of each method were adjusted to dataset features according to the RDP4 manual. A recombination event was accepted only if it was detected by more than two methods with a significance value lower than 0.001 with Bonferroni’s correction, and it was removed from the dataset. The presence of an adequate phylogenetic signal was assessed by likelihood mapping, performed by IQ-tree [51], while the absence of substitution saturation was assessed using the Xia test implemented in DAMBE [52].

Phylogenetic relationships among strains were evaluated using IQ-tree, selecting the substitution model with the lowest Akaike Information Criterion calculated by the software. The reliability of inferred clades was investigated by performing 10000 ultrafast bootstrap replicates. Haplotype networks were calculated using the pegas [53] library in R (Team, 2014).

The migration rates among Italian domestic and wild pig populations were estimated with a discrete trait reconstruction using the Bayesian coalescent-based framework implemented in BEAST 1.8.4. [54]. Molecular clock, population dynamics, and discrete trait substitution models (i.e., symmetric vs. asymmetric migration rate) were selected by evaluation of the Bayesian factor (BF) (i.e., the ratio of the compared model marginal likelihoods, estimated using a Path sampling and Stepping stone approach) as suggested by Baele et al. (2012) [55]. The tree and model parameters were estimated over a 100 million generation Markov Chain Monte Carlo (MCMC) chain, sampling them every 10,000 generations. Run results were accepted only if mixing and convergence (visually inspected using Tracer) were adequate and the estimated sample size (ESS) was higher than 200, after discharging the first 20% generations as burn-in. Parameter estimations were summarized as median and 95HPD. The maximum clade credibility tree was estimated using TreeAnnotator tool of BEAST 1.8.2 package. The BF of well-supported migration rates was calculated using SpreaD3 [56], and a BF greater than 3 was considered significant. A similar analysis was performed including both sampling region and host as discrete traits.

Additionally, a collection of all available PCV-2 ORF2 sequences was downloaded from GenBank. Country, host, and year of sampling were recorded when available. In order to obtain a smaller but still representative dataset, the full sequence database was reduced by clustering all sequences with a pairwise genetic distance lower than 99% using CD-HIT [57] and keeping only one representative sequence. The relationships among Italian and foreign strains were investigated by phylogenetic analysis as previously described.

## Supplementary Materials

The following are available online at https://www.mdpi.com/2076-0817/9/3/221/s1, Figure S1: Phylogenetic tree based on a dataset of ORF2 sequence obtained for worldwide collected PCV-2 strain.
