# Supplementary figures and images for: Free to Circulate: An Update on the Epidemiological Dynamics of Porcine Circovirus 2 (PCV-2) in Italy Reveals the Role of Local Spreading, Wild Populations, and Foreign Countries
Source: Pathogens. 2020 Mar 17;9(3):221. doi: 10.3390/pathogens9030221 (PMC7157736; doi:10.3390/pathogens9030221)

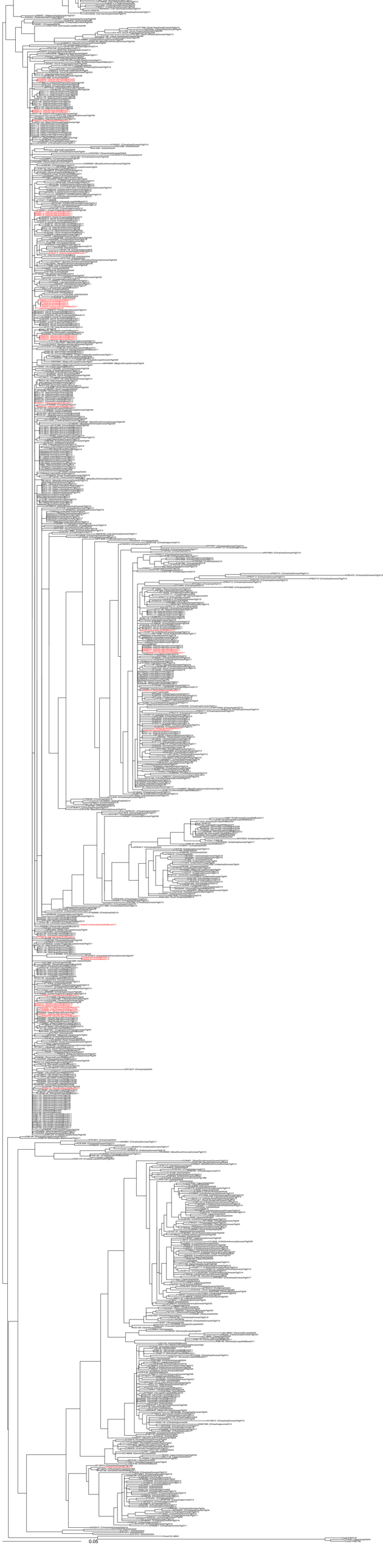

Supplement: Supplementary file 1 [file pathogens-09-00221-s001.pdf]
